# Radical Flexibility and Relationality as Responses to Education in Times of Crisis

**DOI:** 10.1007/s42438-020-00196-3

**Published:** 2020-10-02

**Authors:** George Veletsianos, Shandell Houlden

**Affiliations:** School of Education & Technology, Royal Roads University, Victoria, Canada

**Keywords:** Radical flexibility, Flexible learning, Online learning, Education in crisis

## Abstract

As educational institutions negotiate numerous challenges resulting from the current pandemic, many are beginning to wonder what the future of education may look like. We contribute to this conversation by arguing for flexible education and considering how it can support better—more equitable, just, accessible, empowering, imaginative—educational futures. At a time of historical disorder and uncertainty, we argue that what we need is a sort of radical flexibility as a way to create life-sustaining education, not just for some, but for all, and not just for now, but far into the future. We argue that such an approach is relational, and centers justice and trust. Furthermore, we note that radical flexibility is systemic and hopeful, and requires wide-ranging changes in practices in addition to the application of new technologies.

## Introduction

We are living in times of multiple and multiplying crises, some apparently slow and later, and maybe abstract, others fast and tangible and now. In the immediacy of yesterday and today, the novel coronavirus moves quickly through some of our communities, unevenly striking folks down, disproportionately killing elderly kin, essential workers, and Black people, Indigenous people, and people of color (BIPOC). In the future, or in a place that feels like somewhere else, somewhere at least a little bit distant for now, until it does not, the crisis of the climate emergency, of biodiversity loss and extinction, of people displaced due to climate catastrophe, and of ecological collapse, moving at a pace that for many somehow registers as never too late to address—or else, something yet to be addressed, no doubt just in time.

These two crises—the current pandemic and the climate emergency—appear to many to be distinct from each other, to be separate disasters moving at different speeds in different places. It seems that one can be addressed now, and the other later. But there is no coronavirus pandemic, at least not as we are seeing it today, without the same activities at the root of ongoing and increasingly dire climate disasters (Kolinjivadi 2020), those anthropogenically induced planetary changes so vast as to necessitate their own geologic category of the Anthropocene (Crutzen 2002). The Anthropocene—the epoch characterized by the significance of human impact on our planet—is everywhere, including in our educational institutions, which are negatively impacted by the disasters that now characterize our world, while these same institutions simultaneously fail in fundamental ways to adequately grapple with their role in its perpetuation. What this moment demands of higher education researchers, practitioners, and administrators is meaningful and just response. That response must be manifested in a willingness to navigate and adapt to unpredictable and shifting circumstances that impact people in profoundly uneven ways. It must be about imagining, and then enacting, better futures—meaning imaginative, equitable, accessible, sustainable, and decolonial—for higher education. Such a response needs to be deeply flexible, and flexible across social, cultural, and material differences.

This need has been made painfully clear throughout the far-reaching disruptions flowing from the coronavirus pandemic. The topic of flexible education—that is education that is responsive to learner and societal needs, available in multiple formats, through multiple delivery modes, in multiple timeframes and locations—has perhaps never been so salient, so immediately tangible to the lives of so many people. As various degrees of lockdown in places across the world have closed brick and mortar doors at educational institutions of all levels and kinds, and educational institutions negotiate numerous challenges, many researchers and commentators are turning their attention to the future of education, pondering what it might look like (e.g., Selwyn and Jandrić 2020; Walsh 2020; and Witze 2020 among many others). In this paper, we contribute to the conversation about the future of education at this particular moment, by arguing for radical flexibility and considering how it can support better—more equitable, just, accessible, empowering, imaginative—educational futures. To examine these issues with particular attention to flexible digital education at a time of historical disorder and uncertainty, we draw on our shared knowledges and experiences as educators and non-Indigenous researchers located in the settler colonial context of North America, where one of us is well established in his field of education and learning technologies, and one of us has recently completed her doctoral degree in cultural studies. We proceed by describing the context in which we situate our analysis. Next, we present a theoretical framework to guide our analysis of radical flexibility, and finally, we discuss how radical flexibility may look like in practice, drawing attention to issues of trust and relationality.

## Context

Prior to the pandemic, the anticipated or predicted future of education was already often described as flexible, or as needing to be flexible (Barnett 2014; Gordon 2014). This question of flexibility has developed in itself as a field of inquiry, with increasing attention paid to it in the previous 10 years (Houlden and Veletsianos 2019, 2020; Selwyn 2011; Sheail 2018), though related research has been ongoing for decades (e.g., Daniel 1998; Edwards 1997; Evans 2000; Veletsianos and Houlden 2019). No longer thought simply in terms of access to education at ‘anytime’ from ‘anywhere’, the breadth of how we might make education more flexible has expanded in scope to include everything from shifts in entrance and completion requirements (e.g., flexible admissions policies via prior-learning assessment), and multiple modes of access that provide learners with hybrid choices between in-person and online learning, as well as choice in curriculum and assessment better suited to learners’ needs, for example. In other words, beyond the conventional interpretation of flexibility as being about time and space, flexibility has come to be understood as about making many educational practices malleable and responsive to students and markets (Naidu 2017). Today, the overall ideals of flexible education are to increase the student-centered and empowering aspects of education, thereby improving not just access, but also equity, diversity, inclusion, retention, completion, and satisfaction (Houlden and Veletsianos 2019).

Still, flexible and online education is not without its critics and cautions, and this too has become more apparent, and more widely discussed, currently as the effects of the pandemic reverberate through every aspect of our education systems. Many practitioners for example have recently argued that online learning comes with a whole host of drawbacks, including concerns around accessibility, security, and quality (Fain 2019; Herman 2020; Xie et al. 2020), while researchers have often noted that inequities and technological determinism beleaguer the field of digital education (e.g., Reich in press; Veletsianos 2020). But what is clear to many scholars studying online education, especially those who have been studying it prior to the pandemic, is that a distinction needs to be drawn between the education that was delivered in the spring and summer of 2020, or what has aptly been called emergency remote teaching, and the skillful and well-researched methods of online education (Hodges et al. 2020). What we are bearing witness to now—not just the rapid transition in February and March of 2020, but also the use of online and hybrid options for Fall 2020 and beyond—is flexible digital education deployed in haste, driven by an immediate need to adapt to rapid changes in delivery, namely as suddenly other than face-to-face, all amidst the threat and uncertainty of a widely circulating, poorly understood pathogen.

It is in this specific ongoing context, set against the backdrop of the existential crisis of the climate emergency, that a certain kind of flexible education should emerge, one capable of addressing the crisis at hand and those on the horizon. In this paper therefore, we ask:

- In what do we need to ground this flexibility such that it is capable of responding to the circumstances in which we find ourselves, circumstances which dissolve our false sense of a stable, secure, and reliable future, and underscore the precarity of our globalized infrastructures and networks?
- How to do so without minimizing the under-critiqued and underthought tendencies and mechanisms of flexible education?

## Theoretical Framework

Rather than *begin* with a set of ostensibly flexible solutions (e.g., new platforms or tools) that may either prove unviable or in fact make education more inflexible or less effective for whatever purpose it is intended to serve, institutions require the adoption of a *radical* approach to flexibility. Radical flexibility is not just about making the logistics of education practices easier or more flexible (e.g., providing students with a menu of assignments to choose from), but means taking seriously the nature and purpose of learning itself at the fundamental level of human life, where human life is understood to be enmeshed relationally with all that goes on around, with, and through it. In other words, radical flexibility is a backdoor into thinking not just about how to deliver education equitably, but to ask what kind of education, what kind of university, do we want—which is in turn to ask, what kind of life, what kind of future do we want, and for whom? These are the kinds of questions that education theorists worthy of the crises of the pandemic, climate change, and global racial and colonial injustice are asking (Allen et al. 2020; Bozkurt et al. 2020; Costello et al. 2020). This means that rather than proposing solutions to a series of complex problems, radical flexibility is an invitation to *imagine* and turn to the tools, mechanisms, and systems needed in order to create life-sustaining education, not just for some, but all, and not just for now, but far into the future. Which is to say that radical flexibility is not a structure but is an orientation, one defined by its openness, to how we think about the problems made legible by the pandemic.

To imagine life-sustaining education means beginning with a more just paradigm of who the learner is and can be, or, in other words, that to be flexible is to begin by interrogating assumptions about who the learner is and what tools and capacities they have at their disposable. Elsewhere, we argue (Houlden and Veletsianos 2020) that conventional forms of flexible education that are sometimes reducible to ‘anytime, anyplace’ discourses (i.e., where flexibility is seen through the lens of things such as flexible pacing and the capacity to work from anywhere) are often limited by a structurally implicit orientation to an ideal learning subject, what McMillan Cottom (2015) calls the ‘roaming autodidact’. This is the learner who has the wherewithal to make or access the capital, time, and space for learning in spite of all the other obligations that they have. McMillan Cottom argues that such an orientation inevitably favors white, able-bodied male learners of particular socio-economic status by virtue of the significant privileges that often come with occupying that identity space. Research into the challenges of flexible education for female and BIPOC students, for example, supports this thesis (McMillan Cottom 2017; Selwyn 2011; Simon et al. 2014). In contrast, the imagined ideal learner is the learner as a good liberal humanist subject (Houlden and Veletsianos 2019), he who is independent and above all has fully internalized responsibility as being entirely located in and oriented to the individual (Houlden and Veletsianos 2020). This is the model learner: the one who is self-directed, can command resources, skills, space, and time; the one who has choices and options, and faces far fewer systemic obstacles. This is a learner divorced from the messy and cacophonous reality that the majority of the world faces.

Taking the latter version of the learner to be how we orient our educational systems is to reinforce anti-relational capitalist ideologies and systems that tend to enforce a hierarchy of life according to cis-heteropatriarchic and racial logics (Melamed 2015; Gilmore 2002). It is also to perpetuate structures that disavow the relational nature of life and subjectivity, which is to what radical flexibility has the potential to respond. In other words, instead of developing education for the so-called roaming autodidact, or the learning subject of neoliberal market economics, radical flexibility begins with the principle of the relational nature of all things, a perspective which has a rich and varied theoretical history in a number of critical traditions, including posthumanist thought (e.g., Braidotti 2013; Haraway 2016; Wolfe 2009) and ecofeminist thought (e.g., Gaard 2017; Plumwood 1991), and which was long preceded by Indigenous thought and cultural systems (e.g., Atleo 2011; Todd 2020; Wilson 2008). What unifies some aspects of these diverse perspectives is an ethical orientation guided by an understanding of the relational nature of existence, where all of us are reliant upon and thus responsible to the beings and the worlds in which we live.

With this relational ethical frame in mind, radically flexible education is grounded on the recognition that all learners are embedded in multiple communities and webs of obligations and shared responsibilities that figure deeply into any learning such an individual can do. This kind of education takes its learners to be rich, and complex beings, with deep inter-generational histories of both joy and suffering that impact how and what they both desire and need to learn. To be clear, the responsibility that shapes radical flexibility is in distinct contrast to neoliberal or biopolitical forms of responsibility to which the roaming autodidact is normatively oriented. This latter form of responsibility, seen through Foucault’s (2007) insight into the ways in which governable subjects are taught to internalize their circumstances as wholly their own responsibility, alludes to the learner as being *responsibilized*. The responsibilized learner is the individual who accepts their need for growth and education as a responsibility they have to perform, a duty even, and in a way that meets the narrow parameters of their own already circumscribed desires (Peters 2005). In doing so, they sustain dehumanizing neoliberal logics that assert that the individual is a distinct, self-determining unit, and that their social and economic status is strictly determined through their own actions rather than through systemic factors that constrain or support their activity (Houlden and Veletsianos 2020).

In contrast, radical flexibility approaches responsibility in a far more holistic sense, where responsibility is moved first by the capacity to respond in a life-sustaining and life-supporting way, whether that be to respond to one’s own fundamental needs and desires, the needs and desires of one’s community and broader ecological environment, and even the needs and desires of one’s ancestral and future kin, human and otherwise. This is responsibility understood as a tending of relations. As Potowatomi scholar Whyte (2013: 518) explains:

to be in a relationship is to have responsibilities toward the others in the relationship. Responsibilities refer to the reciprocal (though not necessarily equal) attitudes and patterns of behavior that are expected by and of various parties by virtue of the different roles that each may be understood to play in a relationship.

Systems that inhibit capacities to respond to relationships in this way are actually antithetical to radically flexible education. What’s more, radically flexible education takes its learners to have capacities that shift in meaningful ways throughout the duration of their institutional learning according to the ebbs and flows of everything from their very bodies, to their home lives, to their access to resources, to the effects of violence sustained by broader social and cultural systems that, for example, over-police Black and racialized people, or celebrate white nationalism, or deny the lasting impacts of colonial genocide, or disproportionately harm working class or elderly people, as has so often been the cases with the coronavirus pandemic (Center for Disease Control 2020; Eldeib et al. 2020; García de Müeller et al. 2020). In other words, radical flexibility in education begins with the recognition that learners are relational beings and must be honored and collaborated with as such.

This also means that radically flexible education accounts for present materialities, i.e., it is responsive to the circumstances people live with on a day-to-day basis, why people are doing the work of learning and developing new skills, and who they are doing the work for and with. To begin with, learners understood in this way means flexibility becomes a value or principle that shapes educational infrastructure and pedagogical practices. Here, education is guided by adaptability, suitability, responsiveness, and creativity, all of which fall under the umbrella of justice, or as hooks (1994) calls it, education as the practice of freedom. While some educational technologies may prove beneficial in support of this, they are not *the* solution. They are the means by which flexibility is mobilized and enacted, or how education is made more responsive and more relational. If this core value is obscured—the relational nature of justice—then there is a serious risk of relying on solutions that create more problems than they purport to solve. For example, in the name of permitting students to take exams at home, institutions might insist that they use test-proctoring technology that relies on invasive forms of surveillance, thereby formalizing distrust and deepening dehumanization in our pedagogical methods (Flaherty 2020; Swauger 2020). Those in positions to make decisions—especially administrators such as deans, directors of centers of teaching and learning, and many others in positions of institutional power, and even faculty with power over the technologies they use and lobby for at their institutions—would do well to seriously and continuously consider what problems are being addressed by educational technology interventions, and what values are inherent in the solutions being offered.

## Practice

Key shifts are needed in order to enact this kind of radical flexibility. Bayley (2018: 245) argues that in crises, ‘[w]e need to find practices to stay with the trouble stirred up by late capitalism in the anthropocene moment – a moment where “scholarship committed to the refusal if not the undoing of a world riven by new kinds of warcraft, injustice and exploitation” requires the courage of action’. Such practices are not inseparable from the theory of relationality articulated above, because as hooks (1991): 2) observes:

[w]hen our lived experience of theorizing is fundamentally linked to processes of self-recovery, of collective liberation, no gap exists between theory and practice. Indeed, what such experience makes more evident is the bond between the two – that ultimately reciprocal process wherein one enables the other.

Fundamentally, because radically flexibility is grounded in relationality, it *is* a process of self-recovery and collective liberation. Perhaps to the dismay of some (e.g., educational technology advocates and those who have much to gain from the expanded use of technology in education, ranging from Silicon Valley startups to educational technology consultants), this process, and the practices that come with them, does not by necessity involve inserting new technology into education (though, once again, carefully vetted new technology may prove helpful in cultivating and supporting the approaches we outline here). The shifts radical flexibility may require, however, are not dependent upon anything so facile. What follows are some suggestions into radically flexible education (though no doubt there are many more) which center relationality, both in theoretical and practical terms.

### Trust

To engage with students as relational beings, designers, administrators, and practitioners could consider eliminating the mechanisms and ideologies that reinforce the institution's and learner-educator’s suspicion of the learner (Fawns and Ross 2020). To ground education on the notion that learners must prove themselves is potentially dehumanizing and reduces all the complexities addressed above to a footnote to how learners are expected to participate in their learning, rather than as the very means by which they arrive to their learning.

In practice, this means to *trust* learners—which, in abstract terms is something that many can agree upon, but in practical terms may confound or elicit resistance. In practical terms, trust could mean no more doctor’s notes, no more demand for proof that a family member died or that a learner has actually been diagnosed with Covid-19. It could mean accepting digital copies of reference letters and transcripts while building the digital systems to maintain the privacy and security of such documents (which is where educational technology can actually be useful). It could mean developing sustainable and holistic assessment practices, practices such as having students write critical reflections of their own work, which as Stommel (2018) notes, require releasing ‘attachment to accuracy’ and objectivity to ‘give way to a dialogue – one that is necessarily emergent and subjective’. It means avoiding or abandoning technologies that engender distrust (Ross and Macleod 2018), such as plagiarism detection tools. It may also mean considering that certain foundational elements of established practice may be antithetical to trust, and potentially begin the process of reconsidering and rejecting them. There are too many elements to list here, but to illustrate they may include various principles of instructional design, such as designing instruction grounded on predetermined performance objectives or evaluating outcomes around criterion-referenced assessments. This process may be difficult, not only due to sedimentation around practices that have long been recognized as ‘good’, but also because in the face of crisis, relying on familiar tools/approaches can provide comfort and a sense of stability in the face of uncertainty.

Central here is the recognition that dialog is intrinsically relational, and relationality reinforces trust, which is to say that trust is itself an emergent practice. Trust is not something one gives, but something one does, and the reciprocal nature of it means that it works both ways, that both institution and faculty, as well as learners, can practice trust. Trust is not something that can be granted through statements or declarations without meaningful action, which partially explains why so many individuals in the academy are so skeptical of both pandemic reopening plans that do not have realistic attitudes toward health and safety (e.g., Welch 2020), and of equity and diversity statements in the wake of ongoing efforts to dismantle colonization (Doharty et al. 2020) and ongoing anti-police and Black Lives Matter protests (Howard 2020; Melaku and Beeman 2020).

Counterarguments to the educational practices of trust, such as the argument that some of these adjustments mentioned above are unfair to other students in a class, return to the zero-sum scenario in which justice only looks one way, and thus lose track of the relational nature of education. Importantly, relational approaches are not approaches that disavow accountability. Accountability in relational settings multiplies and manifests in non-prescriptive ways, which is to say, ways that are actually accountable to the complex moving parts of education—the learner, the learner-educator, and the broad ecologies and networks in which both of these beings are embedded. Accountability, for example, might be better enacted in terms of collaborative roles learners occupy and are responsible for together, or by emphasizing a learner’s education in relation to their community responsibilities, but always specific to what their role and responsibility are to their specific community.

Trust also means listening to and responding to the needs of learners, based on their experiences as relational beings enacting, but not reducible to, the role of learners. This could mean, for example, building in accessibility through universal design and the understanding that disability is not something to be overcome or to be treated as a deficit as ableist structures would have it, but is instead ‘a valued part of identity’ (Ban 2020). It also reflects another opportunity to do away with the frame of suspicion that demands proof of disability in the cases of less readily apparent disabilities like chronic or mental illness, for example, and instead ‘views students through a holistic lens and trusts students as people who are experts on their own lives rather than assigning expertise to a third party with medical authority’ (Evans et al. 2017: 365). The effect of this will be to reduce the risk inherent to disclosure of disability, as well as to reduce the labor learners are required to put in with respect to being seen and responded to as their needs dictate, which in turn will permit them to put their labor into their learning.

This same shift away from suspicion to trust also needs to occur for educators, faculty and staff. For example, does a faculty member, adjunct instructor, or graduate student working as a teaching assistant prefer or need to teach online rather than face to face during a pandemic? Demanding they provide narrow forms of evidence of immunocompromise for themselves or members of their household, or urging and requesting them to teach face to face as has been the case for many in the USA, is a failure to respect not only the privacy, expertise, and labor of an individual, but also their relational nature. In practice, what radically flexible education may look like is better support for *all* academic workers—many of whom are far more unfavorably resourced and precariously positioned than others, irrespective of location—which includes everything from reasonable and sustainable working hours, support in technical skills and pedagogy development, support for parental leave, and adequate care during times of illness and disability, for example. More broadly still, it means actively dismantling the institutional forces that contribute to illness and disability, like racism, sexism, and transphobia, and given the lack of supports for anti-oppressive pedagogies and practices (e.g., Valcarlos et al. 2020), expanding supports for them, specifically in the context of postdigital efforts. In the case of disability, for example, this means refiguring the ways by which ‘excellence’ is anchored in individualistic notions of self-reliance and independence, given that too often disabled scholars are expected to perform such a circumscribed form of excellence *in spite of* their disabilities (Merchant et al. 2019). This logic of excellence as the purview of the individual, rather than as being a collaborative way of being, is exemplary of the norms of suspicion within anti-relational systems, as excellence here is defined by the notion that one does it alone.

What follows from this is that radical flexibility is a systemic approach. It does not arise solely through the application of new technologies, partnerships with big tech companies, or semi-nouveau ideas like ‘openness’ or ‘upskilling’ or ‘MOOCs’ or ‘learning analytics’ or ‘learning dashboards’. Rather, it means that those with power, namely privileged faculty and administrators—and the institutions they work within—cannot treat one group in a system relationally while managing another as cogs in a machine. This creates a divide between those who are treated as human beings and those who are not, thereby undermining attending to relationality itself.

## Conclusion

What this pandemic makes abundantly clear is the pressing need not just to build resilient and adaptable ways of designing, developing, and delivering education, but also to subvert the marriage of capitalism and postdigital education in order for education to become a place for the practice of freedom. This is where flexible education becomes radical: it is simultaneously practice and politics, even if education has always been both of those things. What this amounts to is an educational environment in which the people participating and supporting education are understood to be and thus treated as holistic beings, and the digital tools used are meant to facilitate the process of enabling and encouraging the complex relationality of each individual learner and their life. Doing so means attending to the reality of larger circumstances in which we can no longer disavow late capitalism’s racist and imperialist environmental impacts (Heglar 2019; Holthaus 2020; McKibben 2020; Nixon 2013), especially as they are bound up with the effects of anthropogenic climate change (e.g., Alexander 2020; Randall and Gray 2019). Consequently, radically flexible education as an orientation to relationality needs to be far more accountable to the history of education itself than conventional education currently is. By ‘history’ here, we are not alluding to the history of the use of technology in education and the lessons embedded within it. Instead, we are referring to the histories of violence in which a vast majority of Western education systems and institutions are imbricated. Such histories include legacies of slavery and white supremacy (Crawley 2018), which still shape and impact access to education along racialized divides (Reece and O’Connell 2016), with white supremacy, which advantages white people while disadvantaging BIPOC, remaining a structural issue across Western academic institutions (Gillborn 2005; Tate and Bagguley 2016). This also includes attending to histories of Indigenous genocide and colonization, and specifically in settler colonial North America, the role of the land grant system enacted through the Morrill Act of 1862, which ‘turned Indigenous land into college endowments’, and which to this day materially sustains many major academic institutions in North America (Lee and Ahtone 2020; Stein 2020). Such histories are important if we are to meaningfully respond to the ongoing legacies of colonization that currently amount to significant inequality for Indigenous and racialized faculty and students in higher education (Henry et al. 2017) and in racial divides within education more broadly. Without centering these histories in our education institutions, a relational orientation to the future, one predicated on the sustainability of life itself on a finitely resourced planet is nearly impossible, given the direct relationship between the history of unsustainability and the academy (Carp 2013), and the relationship between histories of violence and the production of academic knowledge. Pertinent examples of this are readily visible across the academy: consider the discipline of geography, whose extended engagement with militarism has been argued to be directly tied to settler colonialism and white supremacy (Inwood and Bonds 2016), or the discipline of English which has a long colonial history tied to language and canon (Ngũgĩ 1986; Said 1979), or the history of instructional design and technology which is tied to militarism and war (Reiser 2001). This is to say that if radical flexibility begins with the premise of life as relational, that relationality must extend to the awareness that all are materially bound to the earth and the resources drawn from the earth, as well as to each other, and as such, sustainable futures are inherently connected to that reality and the histories and legacies that shape its future.

We are doubtful that the university as it existed before the pandemic was capable of enacting the kind of radically flexible education outlined above in a robust way. With respect to the climate emergency, Carp (2013: 229) questions whether it is even possible for the academy today to become ecologically sustainable, even though he notes the inevitability of change, that ‘we will either help to shape it and learn to ride it, or we will be inundated by it’. But abrupt and likely permanent change has already arrived in the form of the pandemic, a crisis which Hall (2020: 6) argues will not find its solutions in academia, as ‘[t]he capitalist University cannot save us, because it is driven by short-term economic interests, rather than the long-term conditions of life’. But perhaps it is within this crisis that those of us willing to might make something more out of our circumstances, that especially in this darkness and uncertainty, that we might find hope and the strength to change, to reimagine, and collectively bring into being something new in a way that has long been necessary. Solnit (2020) urges us to remember that ‘[o]rdinary life before the pandemic was already a catastrophe of desperation and exclusion for too many human beings, an environmental and climate catastrophe, an obscenity of inequality’, and this was in many ways as true in the halls of education as anywhere else. But she further reminds us that hope ‘offers us clarity that, amid the uncertainty ahead, there will be conflicts worth joining and the possibility of winning some of them’. If, out of this struggle, we ground our hope in attention to the relational nature of the many worlds in which we all live together, then perhaps we can achieve the radical flexibility truly liberatory education deserves.
